# Clinical and economic impact of molecular testing for BRAF fusion in pediatric low-grade Glioma

**DOI:** 10.1186/s12887-021-03069-1

**Published:** 2022-01-03

**Authors:** Juan David Rios, Russanthy Velummailum, Julie Bennett, Liana Nobre, Derek S. Tsang, Eric Bouffet, Cynthia Hawkins, Uri Tabori, Avram Denburg, Petros Pechlivanoglou

**Affiliations:** 1 Child Health Evaluative Sciences, Peter Gilgan Centre for Research and Learning, The Hospital for Sick Children, 686 Bay Street, 11th Floor – L4 East, Toronto, ON M5G 0A4 Canada; 2 Division of Haematology/Oncology, The Hospital for Sick Children, Toronto, Canada; 3 Radiation Medicine Program, Princess Margaret Cancer Centre, University Health Network, Toronto, ON Canada; 4 Department of Pathology, Hospital for Sick Children, Toronto, ON Canada; 5 Institute of Health Policy, Management and Evaluation, Dalla Lana School of Public Health, University of Toronto, Toronto, Canada

**Keywords:** Pediatric low-grade Glioma, Health economics, Cost-effectiveness, Health technology evaluation, Precision medicine, Molecular testing, Pediatric oncology

## Abstract

**Background:**

Treatment personalization via tumor molecular testing holds promise for improving outcomes for patients with pediatric low-grade glioma (PLGG). We evaluate the health economic impact of employing tumor molecular testing to guide treatment for patients diagnosed with PLGG, particularly the avoidance of radiation therapy (RT) for patients with BRAF-fusion.

**Methods:**

We performed a model-based cost-utility analysis comparing two strategies: molecular testing to determine BRAF fusion status at diagnosis against no molecular testing. We developed a microsimulation to model the lifetime health and cost outcomes (in quality-adjusted life years (QALYs) and 2018 CAD, respectively) for a simulated cohort of 100,000 patients newly diagnosed with PLGG after their initial surgery.

**Results:**

The life expectancy after diagnosis for individuals who did not receive molecular testing was 39.01 (95% Confidence Intervals (CI): 32.94;44.38) years and 40.08 (95% CI: 33.19;45.76) years for those who received testing. Our findings indicate that patients who received molecular testing at diagnosis experienced a 0.38 (95% CI: 0.08;0.77) gain in QALYs and $1384 (95% CI: $-3486; $1204) reduction in costs over their lifetime. Cost and QALY benefits were driven primarily by the avoidance of long-term adverse events (stroke, secondary neoplasms) associated with unnecessary use of radiation.

**Conclusions:**

We demonstrate the clinical benefit and cost-effectiveness of molecular testing in guiding the decision to provide RT in PLGG. While our results do not consider the impact of targeted therapies, this work is an example of the value of simulation modeling in assessing the long-term costs and benefits of precision oncology interventions for childhood cancer, which can aid decision-making about health system reimbursement.

**Supplementary Information:**

The online version contains supplementary material available at 10.1186/s12887-021-03069-1.

## Background

Pediatric low-grade glioma (PLGG) is the most common type of childhood brain tumor, with incidence and survival varying by tumor location and grade [1, 2]. Patients with PLGG generally have a favorable 20-year overall survival (OS) of 85 to 96% [1, 3, 4]. Treatment for patients with PLGG may include surgery, chemotherapy, radiation therapy (RT), or targeted therapies, which may be employed either at diagnosis or at progression [5]. Several studies have demonstrated the benefit of RT in improving progression-free survival (PFS) in patients with PLGG [6–11]. However, its use – particularly in young children – can have a substantial negative long-term impact on health outcomes, including cognitive decline, auditory and visual dysfunction, stroke, vascular damage, secondary malignancies, and endocrine deficiencies [9, 12–14]. Furthermore, patients with central nervous system tumors, of which PLGGs are the most prevalent, are among the most expensive to manage [15], especially when RT is included in the treatment regimen [16].

Recently there has been a wave of tumor-specific therapies in pediatric oncology allowing for personalization of care. New precision diagnostics offer a promising approach to sub-classify patients based on their molecular profile, allowing for risk stratification and treatment with precision therapeutics. Lassaletta et al. found that varied subtypes of BRAF aberration in patients with PLGG, BRAF V600E and BRAF-KIAA1549 fusion, have differing survival and progression outcomes compared to wild-type patients with PLGG [17, 18]. Other retrospective studies have identified patients with the BRAF-fusion as a distinct PLGG subset with better PFS and OS [17, 19]. Given the better phenotypic profile of these patients, attempts to mitigate or avoid the toxicities and deleterious long-term sequelae from traditional modalities of treatment, notably RT, may be warranted. Many institutions have implemented molecular testing for patients with PLGG, and the relevance of molecular stratification for RT-sparing approaches in the BRAF-fusion subset of PLGG is broadly accepted [20, 21]. However, it is unknown whether any health and economic benefits of RT avoidance offset the cost of universal molecular testing in patients with PLGG.

The objectives of this study were to: 1) develop a simulation model to describe the clinical course of patients with PLGG, including any short- and long-term effects of RT; and 2) evaluate the clinical and economic impact of using molecular testing to guide RT treatment decisions in patients with PLGG, accounting for long-term outcomes of patients with PLGG with and without BRAF fusion.

## Methods

We conducted a cost-utility analysis to measure the costs and quality-adjusted life-years (QALYs) of adopting molecular testing to inform the use of RT among patients with PLGG. A QALY is a composite outcome that estimates life expectancy weighted by individuals’ quality of life at any given health state they visited throughout their life [22]. Molecular testing was defined as screening glioma tumor samples for BRAF fusion alterations using the NanoString assay (NanoString Technologies, Seattle, WA). In the intervention arm, patients with PLGG received molecular testing to determine BRAF-KIAA1549 fusion status at diagnosis. The decision to radiate post-treatment progression was conditional on the results of the molecular analysis, with RT eligibility only on patients with non-BRAF fusion status. The control group received no molecular testing, thus fusion status in these patients was unknown. We estimated total healthcare costs, QALYs, and life years for both strategies.

Our model incorporated three major assumptions. Firstly, we assumed that molecular analyses can perfectly reveal fusion status [23]. Next, we assumed that the frequency of treatment-related adverse events for current RT practices resembles outcomes for patients with PLGG treated with RT between 1970 and 1986 [14, 24]. Thirdly, we relied on a pre-specified algorithm to define the population that is likely to receive radiation. Briefly, we used similar eligibility criteria described by Cherlow et al. [11]. The eligibility criteria for receiving RT were: patients between the ages of 3 to 21 years old with unresectable progressive, recurrent PLGG (presence of measurable disease was required), following their first progression. Patients younger than 10 years old were required to have received at least one course of chemotherapy.

### Model structure

We assumed a lifetime horizon as the intervention effects are expected to span over the population’s lifetime. Following good research practice in decision modeling, we followed a simulated cohort of 100,000 patients with PLGG from their initial diagnosis and surgery decision until death [25]. The simulated cohort had characteristics that resembled the Hospital for Sick Children (SickKids) institutional PLGG database. Cost and health outcomes were discounted at a rate of 1.5% as recommended by national guidelines [26].

**Fig. 1:**
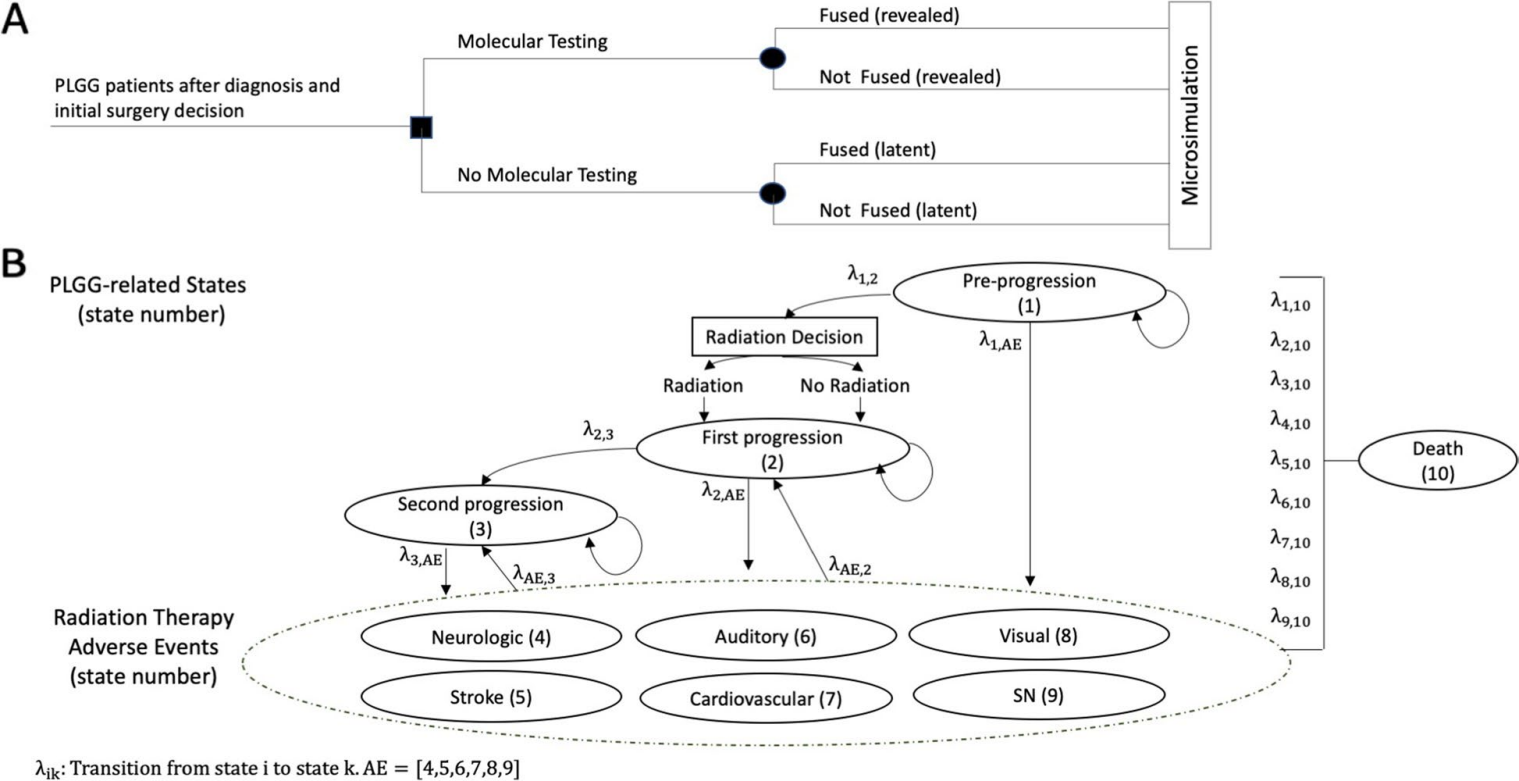
Model description **A** Decision tree for molecular analysis and **B** Microsimulation. Legend: AE; Adverse event, SN; Secondary-neoplasm. Numbers in brackets indicate state number

The effect of molecular testing on long-term healthcare costs and health outcomes for patients with PLGG was estimated using a decision-tree [27] and a 10-state transition microsimulation model [28] (Fig. 1). Patients entered the model after diagnosis and initial surgery decision via: 1) molecular testing to determine BRAF fusion status or 2) no molecular testing. Subsequently, all patients entered into a pre-progression health state. The remaining health states included: first progression, second (or more) progression(s), adverse events (neurological, auditory, visual, stroke, cardiovascular, and subsequent malignant neoplasm), and death. Progression was defined as treatment change related to tumor progression identified via imaging or clinical worsening as outlined previously [18]. We did not include targeted therapies as a possible treatment for PLGG.

### Input parameters

Clinical and molecular data from the Hospital for Sick Children (SickKids) institutional PLGG database from 1987 to 2015 were used to inform the short-term probability of moving between PLGG-related health states. The probability of death and progression were estimated to account for whether an individual had previously progressed. We used a clinically relevant cohort, which excluded patients with a diagnosis of NF1, those treated with RT at first-line therapy, and patients without molecular testing data. We included PLGG patients whose only treatment was surgery. Transition probabilities for long-term treatment-related adverse events were estimated from cumulative incidence curves from the Childhood Cancer Survivorship Study and were a function of age since diagnosis and treatment received. This study, and study protocol as well as a waiver for informed consent were approved by the Research Ethics Board at SickKids (REB # 1000030563).

To adjust for the benefits from RT on PFS, we digitized PFS values from Cherlow et al. [11, 29] with a similar population as our cohort and used the estimated PFS as a proxy for the effect of RT on progression. We conducted analyses varying the benefit of radiation (no RT benefit scenario versus RT benefit scenario) to patients with PLGG. We conducted an additional scenario analysis varying the risk of adverse events due to RT, in 10% increments from a no increased risk due to RT to risk estimates sourced from CCSS. We modeled adverse event-related mortality for secondary neoplasms [30], stroke [31], and cardiovascular events [32]. Treatment-related long-term events were based on the definition from Effinger et al. [14].

We assigned costs from the perspective of a Canadian public healthcare payer. We included costs of PLGG-related treatment (surgery and chemotherapy), and molecular analysis as well as costs associated with long-term patient health outcomes. We incorporated healthcare expenditure unrelated to PLGG to account for future healthcare costs that would be associated with increases in OS. All costs were inflated to 2018 CAD prices using the Statistics Canada Consumer Price Index [33]. Patient-level costs from the Decision Support Department at SickKids, administrative databases, and previously published cost estimates of long-term adverse events were used to inform cost parameters (Supplementary Table 1). Preference-based health-related quality of life measures were retrieved from published literature and calculated from the Canadian Community Health Survey (Supplementary Table 2) [34–36]. To account for the utilities of patients with multiple comorbidities or adverse events, we followed the NICE group’s recommendation of the multiplicative method [37].

All analyses were performed using R [38–42], and published on GitHub (github.com/Pechli-Lab/PLGG-Health-economic-model). We outline the methods used to generate transition probabilities with uncertainty in the Supplementary Methods. Bootstrap methods on 1000 model runs were used to generate uncertainty surrounding utility inputs and to generate confidence intervals for all model outcomes.

## Results

The descriptive characteristics of our institutional PLGG cohort are presented in Table 1. Among the 363 patients with PLGG, 130 (36%) had BRAF-KIAA1549 fusion mutation. Patients with BRAF fusion were diagnosed at a mean age of 9 years old but ranged from 0 to 18 years old at diagnosis and roughly 55% were female. Among patients with BRAF fusion, 2% died and 27% progressed, compared to 7 and 33%, respectively, among those with no BRAF fusion. Kaplan-Meier estimates of PFS and OS for the SickKids institutional cohort are provided in Supplementary Fig. 1, transition probabilities are provided in Supplementary Fig. 2.

**Table 1:** Clinical and Molecular Characteristics of SickKids cohort with PLGG

	All (***N*** = 363)	KIAA1549-BRAF fusion (***N*** = 130)	No Fusion (***N*** = 233)
Age in years at diagnosis, mean (range)	9.8 (0.2–18.5)	9.0 (0.3–17.7)	10.2 (0.2–18.5)
Fused, %	35.81	100.00	0.00
Female, %	49.58	54.62	46.67
Follow up in years, mean (range)			
mortality outcome	8.9 (0.0–30.1)	7.7 (0.0–29.4)	9.6 (0.1–30.1)
progression outcome	5.3 (0.0–22.8)	4.7 (0.0–21.6)	5.6 (0.0–22.8)
Died, N (%)	19 (5.23)	3 (2.31)	16 (6.87)
Progressed, N (%)	113 (31.13)	35 (26.92)	78 (33.48)

**Fig. 2:**
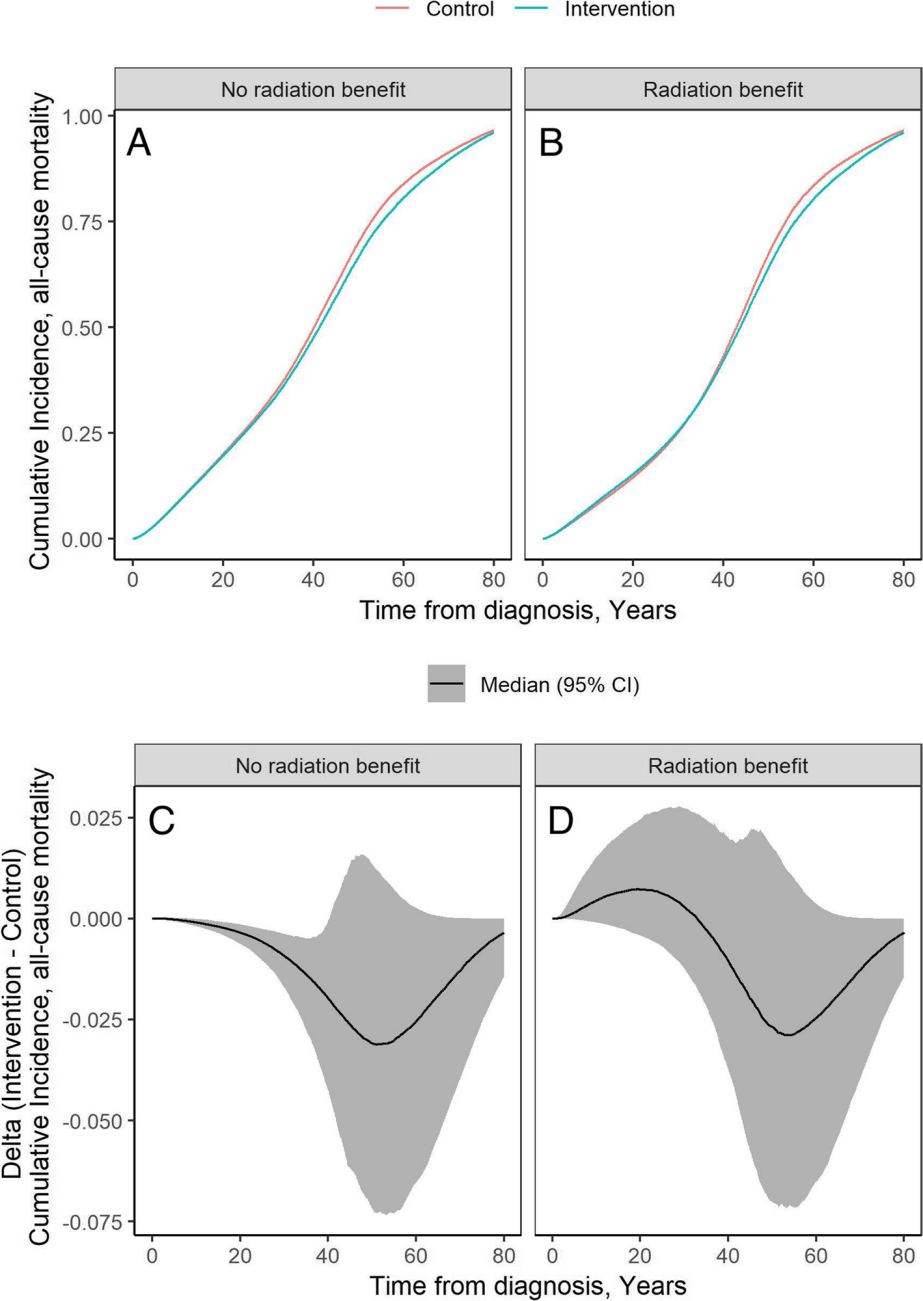
Cumulative incidence of all-cause mortality for all and difference in cumulative incidence of all-cause mortality. Legend: Cumulative incidence (top), difference in cumulative incidence (bottom)

Figure 2 shows the estimated cumulative mortality for those who received molecular testing (intervention) and those who did not (control) under a ‘no RT benefit’ scenario (panel A), as well as under an assumption of RT benefit, derived from Cherlow et al. [11] (panel B). We also demonstrate the mortality difference between molecular testing and no molecular testing under ‘no radiation benefit’ (panel C) and ‘RT benefit’ scenarios (panel D). The 20-year OS after diagnosis in the control arm, assuming no RT benefit, was estimated at 80% for all individuals, and 87.6% for fused patients. Assuming an RT benefit, the 20-year OS in the no molecular testing arm was 85.4, and 90.7% for fused patients.

Table 2 provides discounted estimates of costs, life-years, and QALYs for the testing (intervention) and no-testing (control) strategies, under both radiation-effect scenarios. When no radiation benefit was assumed, patients who received molecular testing at diagnosis experienced a gain in life expectancy of 1.07 (95% CI, 0;2.39) years, 0.38 (0.08;0.77) more QALYs, and a $1384 ($-1204; $3486) reduction in total costs compared to patients who did not receive molecular testing. Even under the assumption of radiation benefit to survival, the testing strategy remained dominant, with a gain in life expectancy of 0.61 (− 0.78;2.25) years, 0.28 (− 0.11;0.72) greater QALY, and $1232 ($-3508; $1938) reduction in costs in the intervention arm. In the no radiation benefit scenario, the life expectancy after diagnosis under molecular testing was 40.08 (33.19;45.76) and 39.01 (32.94;44.38) years under no testing. When assuming a radiation benefit the life expectancy after diagnosis was 42.05 (34.98; 47.29) for those who received testing and 41.44 (35.8;46.09) for those who did not receive testing. Fused patients had higher QALYs and lower total costs compared to non-fused patients when evaluating the testing strategy against the no-testing strategy. In the testing strategy, fused patients had 1.07 (0.23;2.16) and 0.77 (− 0.3;2.01) more QALYs in the no radiation benefit scenario radiation and the radiation benefit scenario respectively. The subgroup that benefited the most from the testing intervention were the fused patients who progressed, since their treatment decisions were modified as a result of the test results. We present detailed outcomes for the subset of fused individuals and fused individuals who progressed in Supplementary Table 3.

**Table 2:** Estimates of life-years after diagnosis, QALYs (Discounted) and Costs (Discounted)

Variable	Intervention	Control	Delta (Intervention-Control)
**No Radiation Benefit**			
Life-years	40.08 (33.19;45.76)	39.01 (32.94;44.38)	1.07 (0;2.39)
QALY	11 (9.16;12.59)	10.62 (9.04;12.03)	0.38 (0.08;0.77)
Total Cost	$215,358 ($159,507; $232,728)	$216,742 ($161,250; $234,069)	$-1384 ($-3486; $1204)
PLGG	$90,839 ($75,023; $108,612)	$90,244 ($74,885; $107,176)	$595 ($106; $2083)
AE	$124,519 ($76,439; $151,351)	$126,498 ($78,418; $153,232)	$-1979 ($-4414; $1017)
**Radiation Benefit**			
Life-years	42.05 (34.98;47.29)	41.44 (35.8;46.09)	0.61 (−0.78;2.25)
QALY	11.46 (9.69;12.91)	11.18 (9.68;12.49)	0.28 (−0.11;0.72)
Total Cost	$217,393 ($157,671; $236,003)	$218,624 ($155,869; $237,155)	$-1232 ($-3508; $1938)
PLGG	$78,899 ($68,624; $91,119)	$75,259 ($66,745; $85,262)	$3640 ($984; $8397)
AE	$138,494 ($81,832; $160,740)	$143,366 ($84,549; $164,863)	$-4872 ($-10,600; $-146)

Estimates reported with 95% Confidence Interval in brackets. All costs are given in Canadian dollars

QALY: Quality-adjusted Life Years; PLGG: Pediatric Low-Grade Glioma; AE: Adverse Events

Supplementary Fig. 3 shows the difference in cumulative all-cause mortality between the intervention and control groups for various subsets. When assuming no radiation benefit (panels A and B), the control and intervention arms have similar mortality until 30 years after diagnosis, after which mortality due to radiation-related adverse events leads to improved survivorship in the intervention arm. When assuming a positive radiation therapy effect (panels C and D), the control arm has improved survival in the short-term due to improved disease control, but cumulative mortality from radiation-related adverse events results in increased mortality relative to the intervention arm by 34 years after diagnosis. Results from scenario analyses varying the radiation related AE risk are presented in Supplementary Table 4.

**Fig. 3:**
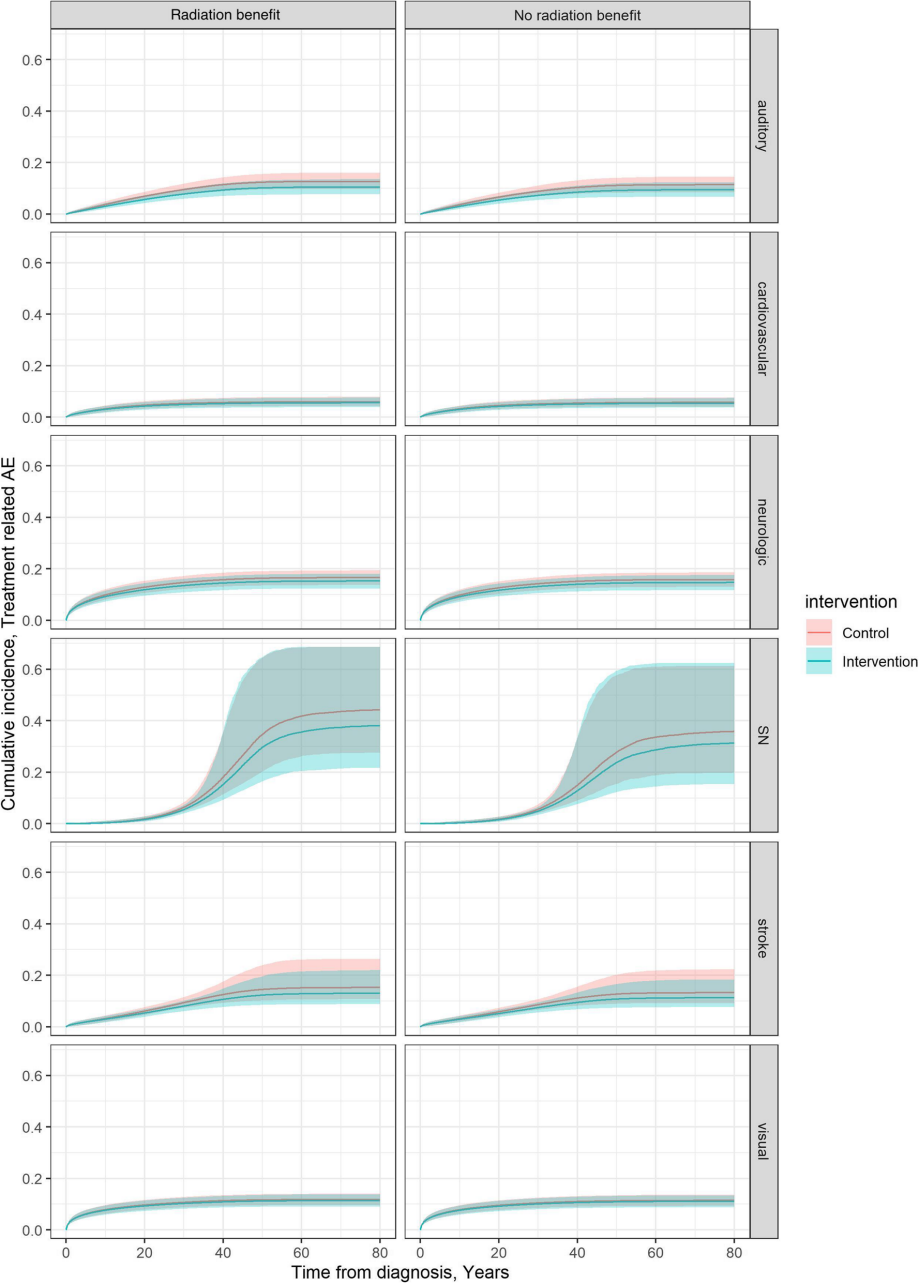
Cumulative incidence of radiation-related adverse events assuming no radiation benefit and with radiation benefit

A principal driver of the differences in QALYs between study arms is the increased incidence of radiation-related adverse events – specifically, auditory, neurologic, stroke, and secondary neoplasms associated with decreases in quality of life (Fig. 3). We provide estimates of cumulative incidence of stroke, secondary neoplasm, and cardiovascular events in Supplementary Fig. 4. The differences in total costs are due to large costs associated with the treatment of radiation-related adverse events. The number needed to test to change a radiation decision was 9.5 (4;13.8) patients with PLGG.

**Fig. 4:**
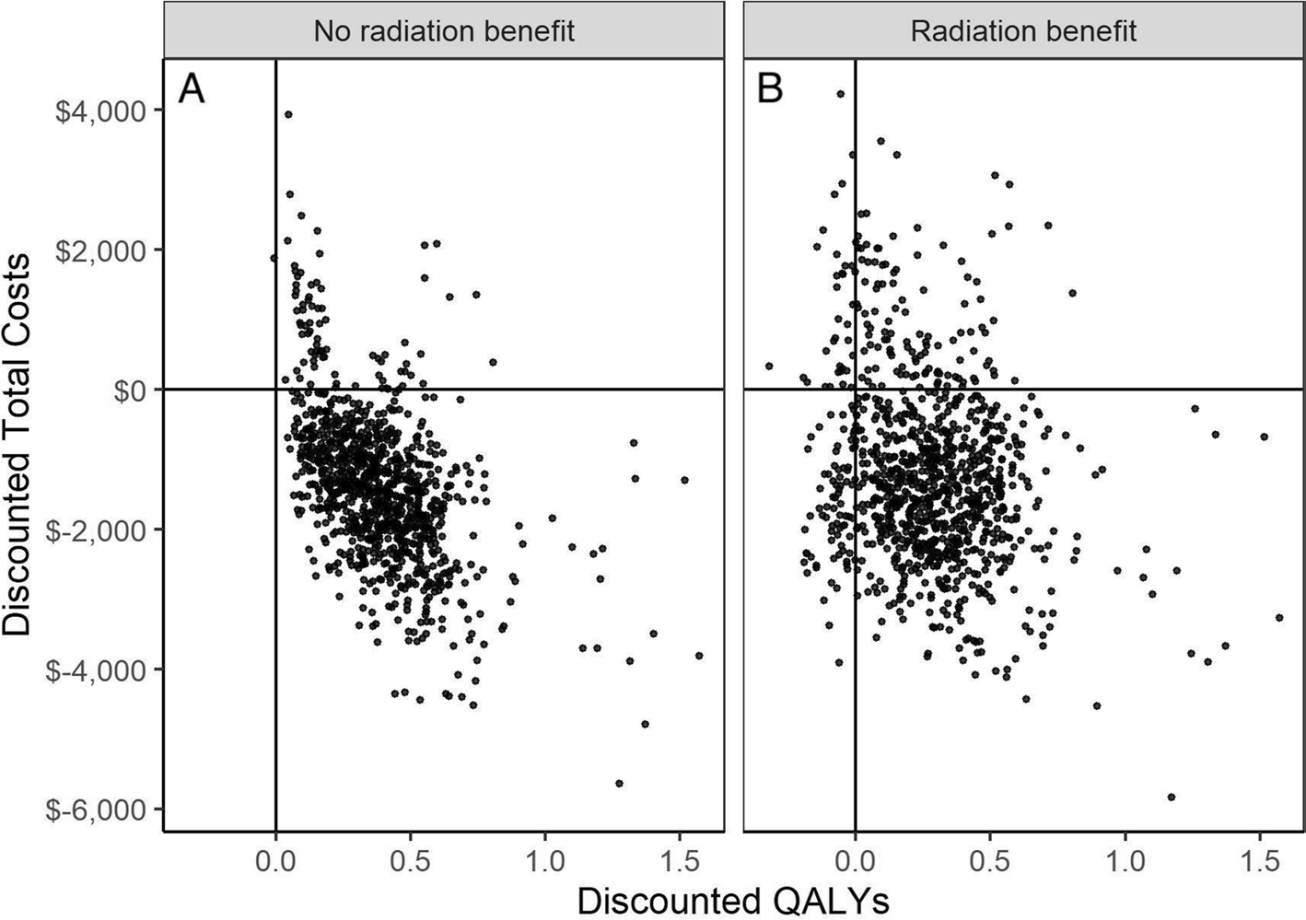
Cost-effectiveness plane indicating the incremental cost and incremental effectiveness estimates related to molecular testing for each probabilistic simulation assuming both **A** a no-radiation benefit and **B** a radiation benefit

In Fig. 4, we plot differences in discounted QALYs and total costs when assuming no-radiation benefit (Panel A) and radiation benefit (Panel B) for all model runs. This figure demonstrates the large underlying uncertainty in the expected benefit attached to molecular testing. This is primarily due to uncertainty of the long-term estimates of treatment-related adverse events.

## Discussion

To our knowledge, this is the first study to evaluate the cost-effectiveness of molecular testing for patients with PLGG. We found that the use of molecular testing in patients with PLGG at diagnosis facilitates treatment decisions – specifically, an RT avoidance strategy – that results in increased QALYs and decreased costs over the life course. These benefits are sustained even when accounting for potential PFS benefits associated with the use of RT in PLGG. Importantly, our findings also confirm previous results demonstrating that patients with PLGG with BRAF fusion have improved PFS and OS as compared with BRAF wild-type patients [17–19].

Our study is significant both for the specific value it ascribes to BRAF fusion analysis in PLGG and for the underlying economic model it developed and tested. We describe a comprehensive life-course approach to evaluating a precision medicine intervention for a prevalent childhood cancer, with potential applications to additional therapeutic questions in PLGG (e.g. targeted therapies) as well as to life-course modeling of the late effects of childhood cancer therapy. Our findings underscore the mounting need for, and value of, economic modeling to evaluate the trade-offs inherent in the adoption of precision cancer technologies.

As health systems grapple with sound stewardship of scarce resources in the face of technological advances, evidence-informed policy on the funding of novel health technologies will prove crucial to system sustainability and equitable access to care. Our study is an early example of the sophisticated modeling required to evaluate innovative diagnostics and therapeutics in childhood cancer; its design is relevant to a range of diseases and treatment paradigms to inform optimal health system resource allocation. Economic models of this nature are of particular value in the evolving precision medicine space, where clinical evidence will derive increasingly from novel trial designs (such as basket trials and adaptive designs) that fragment previously homogenous disease cohorts and challenge accepted hierarchies of evidence [43–45].

The decision to provide radiation therapy is highly heterogeneous across jurisdictions, worldwide [46]. In addition, conducting jurisdiction-specific cost-effectiveness analysis provides decision-makers with more accurate and relevant information, since interventions that are cost-effective in one jurisdiction may not have similar results when re-estimated for another jurisdiction. We have conducted our analysis from the perspective of the Canadian health care system, but researchers from different jurisdictions might wish to adapt our analysis to their own jurisdictions. To facilitate this, we have published the simulation model code in a public repository. Publishing out model code will also facilitate the evaluation of other treatments for PLGG, such as novel targeted therapies.

Our study has several limitations. First, there is considerable uncertainty in some of our parameter estimates related to the incidence of long-term adverse events, benefits of RT, and long-term PLGG related outcomes resulting in large uncertainty in life-course estimates of survival and some RT-related adverse event utilities, namely, cardiac and secondary neoplasms. Second, our approach to modeling RT use in patients with PLGG relies on simplifying assumptions that likely do not adequately capture the variability in real-world practice. Specifically, we modeled radiation decision only at 1st progression while PLGG patients are at risk of receiving radiation in subsequent progressions [47]. We have also modeled the risk of treatment-related adverse events independently from the number of progressions and lines of therapy a patient has experienced. The past several decades have witnessed philosophical shifts in the perceived role of RT in pediatric PLGG, resulting in systemic variations in RT practices across treating institutions. In our cohort, RT was rarely administered to patients with PLGG after the year 2000, due to changes in institutional treatment guidelines; consequently, we lacked data from a direct comparator group to assess the natural history of BRAF fusion status with and without RT. To address this, we simulated a scenario for RT use relying on inclusion and exclusion criteria from an RT clinical trial [11]. In real-world practice, variations from these criteria are likely, hence the population in Cherlow et al. may not be representative of the larger LGG pediatric population [11]. Furthermore, there is no strong evidence of whether the benefit of RT varies by BRAF fusion status. We also do not consider the impact of the use of proton therapy on the cost-effectiveness of BRAF fusion testing. Lastly, estimates of the late effects of RT data are from patients with PLGG who had RT between 1970 and 1986 [14]. This likely reflects the upper bound estimate of the risk of RT given improvements in RT since the CCSS cohort received treatment [46]. Scenario analysis results indicate the benefits of BRAF fusion testing even under assuming a conservative risk of radiation related adverse events.

## Conclusions

Our study findings show the clinical benefit and cost-effectiveness of molecular testing in guiding the decision to provide RT to patients with PLGG, a prevalent childhood cancer. This is highly relevant given current practice where RT is still prevalent in the treatment of PLGG [46]. Our model in these patients can be extended to address personalized therapeutic decisions, including the evolving use of BRAF-targeted agents for disease management. The rigorous simulation model developed in this study with the incorporation of late effects and survivorship utilities is a useful way to evaluate precision medicine innovations and can be used to inform optimal health system resource allocation in childhood cancers.

## Supplementary Information

**Additional file 1.** Supplementary Material. Supplementary methods and tables.

## Data Availability

The datasets used and/or analysed during the current study are available from the corresponding author on reasonable request.

## Abbreviations

CAD: Canadian Dollars
CI: Confidence intervals
OS: Overall survival
PFS: Progression-free survival
PLGG: Pediatric low-grade glioma
QALY: Quality-adjusted life years
RT: Radiation therapy
