# Supplementary material for: Clinical and economic impact of molecular testing for BRAF fusion in pediatric low-grade Glioma
Source: BMC Pediatr. 2022 Jan 3;22:13. doi: 10.1186/s12887-021-03069-1 (PMC8722113; doi:10.1186/s12887-021-03069-1)
Supplement: Supplementary file 1 — Additional file 1. Supplementary Material. Supplementary methods and tables. [file 12887_2021_3069_MOESM1_ESM.docx]

**Additional File 1**

**Supplementary Methods**

Sourcing treatment-related adverse events:

The probability of having a treatment-related adverse event (AE) was sourced from a retrospective cohort study of 5-year survivors of astrocytoma who were diagnosed between 1970-1986.^1^The retrospective study followed 1,182 astrocytoma survivors for up to 30 years after diagnosis. Most of the treatment received by patients with astrocytoma included upfront surgery and radiation. The retrospective study estimated the cumulative incidence of neurologic, auditory, visual, stroke, cardiac, subsequent neoplasm, and endocrine AEs stratified by radiation exposure. The Supplementary material in Effinger et al.^1^ outlines the description of each AE. The cumulative incidence curves were digitized, and patient-level data were reconstructed using the methods described in Guyot et al.^2^

Estimating transition probabilities with uncertainty:

To estimate the probability of having a treatment-related adverse or PLGG-related transition, we created 1,000 bootstrapped samples of the patient-level data. For each transition probability, we fit six survival distributions (gamma, exponential, Weibull, Gompertz, log-logistic, and log-normal) to each bootstrapped sample. The best-fitting distribution was selected according to Akaike Information Criterion (AIC), a statistical measure of fit. We then extrapolated estimates to model the probability of an event occurring past the maximum follow-up of the bootstrapped patient-level data. The outcome of this is 1,000 extrapolations of the best-fitting survival distribution based on bootstrapped data for each possible transition.

Our approach to estimating transition probability has several advantages, primarily it does not rely on a singular survival distribution but a mixture of distributions according to the bootstrapped data. Secondly, it respects any underlying correlation between transitions, for example, if a bootstrapped sample has especially poor outcomes for BRAF Fused patients this will be reflected in the transition probabilities associated with progression and mortality.

There are also several limitations to this approach. The first is that we assume that the long-term radiation outcomes for patients with astrocytoma who were diagnosed between 1970- 1986 represents the outcomes for all PLGG patients radiated today. We are also uncertain about extrapolating outcomes beyond the maximum follow-up of patient-level data which is presented by the large 95% confidence intervals in **Supplementary Figure 2.**

**Supplementary Methods References**

1. Effinger KE, Stratton KL, Fisher PG, et al. Long-term health and social function in adult survivors of paediatric astrocytoma: A report from the Childhood Cancer Survivor Study. *Eur J Cancer*. 2019;106(December 2018):171-180. doi:10.1016/j.ejca.2018.10.016’
2. Guyot P, Ades AE, Ouwens MJNM, Welton NJ. Enhanced secondary analysis of survival data: Reconstructing the data from published Kaplan-Meier survival curves. *BMC Med Res Methodol*. 2012. doi:10.1186/1471-2288-12-9

**Supplementary Tables**

| Type | State | Time/Cycles | Value | Source |  |
| --- | --- | --- | --- | --- | --- |
| PLGG related - Inpatient costs | Pre-progression | Cycle 1 | $ 14,955.52 | The Hospital for Sick Children Decision Support |  |
|  |  | Cycle 2 until patient is 18 years old | $ 357.26 |  |  |
|  | Progression one | Cycle 1 | $ 3,876.69 | The Hospital for Sick Children Decision Support |  |
|  |  | Cycle 2 until patient is 18 years old | $ 806.40 |  |  |
|  | Progression 2+ | Cycle 1 | $ 7,358.42 | The Hospital for Sick Children Decision Support |  |
|  |  | Cycle 2 until patient is 18 years old | $ 1,367.02 |  |  |
|  | Death | 12 Cycles before death | $ 25,960.21 | ^1^ |  |
| PLGG related -Outpatient costs | Pre-progression | Cycles 1-17 | $ 3,823.31 | The Hospital for Sick Children Finance Department |  |
|  | Progression one | Cycles 1 to 13 (0-52 weeks after progression) | $ 3,786.90 |  |  |
|  | Progression 2+ | Cycles 1 to 13 (0-52 weeks after progression) | $ 3,786.90 |  |  |
| Treatment Related Adverse Events | Neurologic | Cycle 1-2 post-injury | $30,500 ($28,900-$32,100) | ^2^ |  |
|  |  | Cycles 3 to 11 after injury | $7,400 ($6,700-$8,200) |  |  |
|  |  | Cycles 12 to (5 cycles before death) | $1,500 ($1,200-$1,700) |  |  |
|  | Auditory | Cycle 1 to death | $ 8.33 | ^3^ |  |
|  | Visual | Cycles 1 to 12 | $ 17.95 | ^4^ |  |
|  |  | Cycles 13 to 24 | $ 8.98 | ^4^ |  |
|  |  | Cycles 25 to death | $ 5.98 | ^4^ |  |
|  | Stroke | Cycle 1 | $ 21,735.00 | ^5^ |  |
|  |  | Cycles 2 to 20 | $ 4,933.00 | ^5^ |  |
|  | Cardiac | Cycle 1 | $ 20,565.00 | ^5^ |  |
|  |  | Cycles 2 to 21 | $ 2,997.00 | ^5^ |  |
|  | Subsequent Neoplasm | Pre-diagnosis (3 cycles) | $ 2,842.71 | ^6^ |  |
|  |  | Cycles 1 - 6 | $ 4,566.84 | ^6^ |  |
|  |  | Cycles 7- (12 cycles before death) | $ 664.50 | ^6^ |  |
|  |  | 12 cycles before death (Terminal) | $ 5,494.93 | ^6^ |  |
| Molecular Testing Costs | NanoString Test | One-time cost | $ 350.00 | C. Hawkins Team |  |
|  | FISH (for small tissue) | One-time cost | $ 500.00 | C. Hawkins Team |  |
| National Government health expenditure per person | By Age group | <1-4 | $ 198.61 | Table E.1.19.1^7^;  with 2016 Population from Table 17-10-0005- 01 ^8^ |  |
|  |  | 5-9 | $ 117.72 |  |  |
|  |  | 10-14 | $ 123.87 |  |  |
|  |  | 15-19 | $ 153.85 |  |  |
|  |  | 20-24 | $ 162.95 |  |  |
|  |  | 25-29 | $ 195.81 |  |  |
|  |  | 30-34 | $ 218.25 |  |  |
|  |  | 35-39 | $ 211.98 |  |  |
|  |  | 40-44 | $ 209.87 |  |  |
|  |  | 45-49 | $ 234.96 |  |  |
|  |  | 50-54 | $ 273.42 |  |  |
|  |  | 55-59 | $ 329.07 |  |  |
|  |  | 60-64 | $ 407.10 |  |  |
|  |  | 65-69 | $ 542.05 |  |  |
|  |  | 70-74 | $ 703.22 |  |  |
|  |  | 75-79 | $ 942.80 |  |  |
|  |  | 80-84 | $ 1,306.34 |  |  |
|  |  | 85-89 | $ 1,938.94 |  |  |
|  |  | 90+ | $ 2,455.38 |  |  |
| Auditory cost was sourced as the coverage by the Assistive Devices Program (ADP) in Ontario of 75% of the average cost of a hearing aid in Canada (up to $500) over 5 years (average lifespan of Hearing Aid)  Subsequent Neoplasms were defined as brain, other tumour sites, melanoma, female breast, lymphoma and leukemia ^9^  Cycle refers to a 1-month period in the economic model.  Assumed Outpatient chemotherapy costs at 1^st^ line for Vinblastine, 2^nd^ line for Vincristine + Carboplatin, and 3^rd^ line for Thioguanin, Procarbazine, Lomustinem Vincristine (TPCV). Chemotherapy costs included allied health, lab costs, quality control, care coordination/follow-up & other indirect costs. | | | | | |

**Supplementary Table 1**: Costs Included in the Economic Model

**Supplementary Table 1 References**

1. Oliveira C de, Bremner KE, Liu N, et al. Costs of cancer care in children and adolescents in ontario, canada. Pediatric blood & cáncer. 2017;64(11):e26628.
2. Chan BC-F, Cadarette SM, Wodchis WP, Krahn MD, Mittmann N. The lifetime cost of spinal cord injury in ontario, canada: A population-based study from the perspective of the public health care payer. The journal of spinal cord medicine. 2019;42(2):184-193.
3. Assistive Devices Program. Policies and Procedures Manual for the Assistive Devices Program May 2016.; 2016.
4. Leung VC, Pechlivanoglou P, Chew HF, Hatch W. Corneal Collagen Cross-Linking in the Management of Keratoconus in Canada: A Cost-Effectiveness Analysis. Ophthalmology. 2017;124(8):1108-1119. doi:[10.1016/j.ophtha.2017.03.019](https://doi.org/10.1016/j.ophtha.2017.03.019)
5. Tawfik A, Wodchis WP, Pechlivanoglou P, Hoch J, Husereau D, Krahn M. Using Phase-Based Costing of Real-World Data to Inform Decision–Analytic Models for Atrial Fibrillation. Applied Health Economics and Health Policy. 2016;14(3):313-322. doi:[10.1007/s40258-016-0229-2](https://doi.org/10.1007/s40258-016-0229-2)
6. Oliveira C de, Pataky R, Bremner KE, et al. Phase-specific and lifetime costs of cancer care in Ontario, Canada. BMC Cancer. 2016;16(1):1-12. doi:[10.1186/s12885-016-2835-7](https://doi.org/10.1186/s12885-016-2835-7)
7. Canadian Institute for Health Information. National Health Expenditure Trends, 2016 | CIHI. Published online 2018. Accessed March 5, 2020. <https://www.cihi.ca/en/national-health-expenditure-trends-1975-to-2019>
8. Statistics Canada. Table 17-10-0005-01 Population estimates on July 1st, by age and sex. Published online 2016. Accessed March 5, 2020. <https://www150.statcan.gc.ca/t1/tbl1/en/tv.action?pid=1710000501>
9. Effinger KE, Stratton KL, Fisher PG, et al. Long-term health and social function in adult survivors of paediatric astrocytoma: A report from the Childhood Cancer Survivor Study. European Journal of Cancer. 2019;106(December 2018):171-180. doi:[10.1016/j.ejca.2018.10.016](https://doi.org/10.1016/j.ejca.2018.10.016)
10. Chan BC, Cadarette SM, Wodchis WP, Krahn MD, Mittmann N. The lifetime cost of spinal cord injury in Ontario, Canada: A population-based study from the perspective of the public health care payer. The journal of spinal cord medicine. 2019 Mar 4;42(2):184-93.

| Health State | Age Group | Utility | | Source |
| --- | --- | --- | --- | --- |
|  |  | Mean Estimate | Uncertainty (SE) |  |
| Adult Survivors PLGG | Adults | 0.809 | 0.00255 | ^1^ |
| Children with PLGG | Children | 0.8671 | 0.02409 | ^2^ |
| Cancer with no Adverse Events | Adults | 0.8211 | 0.00311 | ^3^ |
| General population with no Adverse Events | Adult | 0.8915 | 0.00066 | ^3^ |
| General population Ages 12-19 | Children | 0.8860 | 0.00255 | ^4^ |
| Death | All | 0 |  | Assumed |
| Neurologic | Adults | 0.3200 | 0.02301 | ^5^ |
| No Neurologic | Adults | 0.8740 | 0.00070 | ^3^ |
| Auditory | Children | 0.707 | 0.03838 | ^6^ |
| Auditory | Adults | 0.6056 | 0.00484 | ^3^ |
| No Auditory | Adults | 0.8799 | 0.00069 | ^3^ |
| Visual | Adults | 0.8 | 0.03656 | ^7^ |
| No Visual | Adults | 0.8750 | 0.00070 | ^3^ |
| Stroke | Adults | 0.5740 | 0.01003 | ^3^ |
| No Stroke | Adults | 0.8740 | 0.00070 | ^3^ |
| Cardiac | Adults | 0.7590 | 0.00090 | ^8^ |
| No Cardiac | Adults | 0.8781 | 0.00068 | ^3^ |
| Subsequent Neoplasms - Diagnosed 0-5 years | Adults | 0.7609 | 0.00634 | ^3^ |
| No Subsequent Neoplasms - Diagnosed 0-5 years | Adults | 0.8729 | 0.00071 | ^3^ |
| Subsequent Neoplasms - Diagnosed 5+ years | Adults | 0.8073 | 0.00359 | ^3^ |
| No Subsequent Neoplasms - Diagnosed 5+ years | Adults | 0.8759 | 0.00072 | ^3^ |
| Abbreviations: Var. Variance  Acronyms: SE: Standard Error, CI: Confidence Interval | | | | |

**Supplementary Table 2:** Utilities Included in the Economic Model

**Supplementary Table 2 References**

1. Yeh JM, Hanmer J, Ward ZJ, et al. Chronic Conditions and Utility-Based Health-Related Quality of Life in Adult Childhood Cancer Survivors. *Journal of the National Cancer Institute*. 2016;108(9). doi:[10.1093/jnci/djw046](https://doi.org/10.1093/jnci/djw046)

2. Barr RD, Simpson T, Whitton A, Rush B, Furlong W, Feeny DH. Health-related quality of life in survivors of tumours of the central nervous system in childhood - A preference-based approach to measurement in a cross-sectional study. *European Journal of Cancer*. 1999;35(2):248-255. doi:[10.1016/S0959-8049(98)00366-9](https://doi.org/10.1016/S0959-8049(98)00366-9)

3. Statistics Canada. Canadian Community Health Survey 2009/2010, 2013/2014, 2015/2016. Published online 2016.

4. Guertin JR, Feeny D, Tarride JE. Age- and sex-specific Canadian utility norms, based on the 2013-2014 Canadian Community Health Survey. *Cmaj*. 2018;190(6):E155—-E161. doi:[10.1503/cmaj.170317](https://doi.org/10.1503/cmaj.170317)

5. Post PN, Stiggelbout AM, Wakker PP. The utility of health states after stroke a systematic review of the literature. *Stroke*. 2001;32(6):1425-1429. doi:[10.1161/01.STR.32.6.1425](https://doi.org/10.1161/01.STR.32.6.1425)

6. Smith-Olinde L, Grosse SD, Olinde F, Martin PF, Tilford JM. Health state preference scores for children with permanent childhood hearing loss: A comparative analysis of the QWB and HUI3. *Quality of Life Research*. 2008;17(6):943-953. doi:[10.1007/s11136-008-9358-x](https://doi.org/10.1007/s11136-008-9358-x)

7. Leung VC, Pechlivanoglou P, Chew HF, Hatch W. Corneal Collagen Cross-Linking in the Management of Keratoconus in Canada: A Cost-Effectiveness Analysis. *Ophthalmology*. 2017;124(8):1108-1119. doi:[10.1016/j.ophtha.2017.03.019](https://doi.org/10.1016/j.ophtha.2017.03.019)

8. Stevanović J, Pechlivanoglou P, Kampinga MA, Krabbe PFM, Postma MJ. Multivariate meta-analysis of preference-based quality of life values in coronary heart disease. *PLoS ONE*. 2016;11(3):1-20. doi:[10.1371/journal.pone.0152030](https://doi.org/10.1371/journal.pone.0152030)

| **Subset of fused patients** | | | | |
| --- | --- | --- | --- | --- |
| **No Radiation Benefit** | | | | |
| **Variable** | **Intervention** | **Control** | **Delta (Intervention-Control)** | |
| Life-years | 49.32 (36.23;57.62) | 46.33 (35.91;53.04) | 2.99 (0;6.68) | |
| QALY | 13.72 (10.24;16.03) | 12.65 (9.93;14.51) | 1.07 (0.23;2.16) | |
| Total Cost | $217,468 ($156,617;$242,546) | $221,571 ($160,421;$246,054) | $-4,103 ($-9,878;$3,122) | |
| PLGG | $77,581 ($62,377;$102,659) | $76,160 ($62,186;$97,136) | $1,421 ($58;$5,584) | |
| AE | $139,887 ($78,328;$173,061) | $145,411 ($82,676;$175,981) | $-5,524 ($-12,335;$2,880) | |
| **Radiation Benefit** | | | | |
| Life-years | 49.32 (36.23;57.62) | 47.61 (37.78;53.33) | 1.7 (-2.18;6.26) | |
| QALY | 13.72 (10.24;16.03) | 12.95 (10.35;14.59) | 0.77 (-0.3;2.01) | |
| Total Cost | $217,446 ($156,674;$242,545) | $221,122 ($155,329;$244,931) | $-3,676 ($-9,990;$5,310) | |
| PLGG | $77,586 ($62,378;$102,633) | $67,664 ($59,271;$80,480) | $9,922 ($2,493;$23,192) | |
| AE | $139,860 ($78,331;$173,129) | $153,458 ($88,268;$178,898) | $-13,597 ($-29,599;$-349) | |
| **Subset of fused patients who progressed** | | | | |
| **No Radiation Benefit** | | | | |
| **Variable** | **Intervention** | **Control** | | **Delta ( Intervention-Control)** |
| Life-years | 43.18 (26.11;57.59) | 36.34 (24.4;45.35) | | 6.83 (0;12.9) |
| QALY | 12.09 (7.57;15.89) | 9.64 (6.67;11.88) | | 2.45 (0.58;4.1) |
| Total Cost | $238,974 ($188,317;$264,719) | $248,824 ($193,374;$273,604) | | $-9,849 ($-21,779;$6,732) |
| PLGG | $122,209 ($88,115;$181,323) | $119,076 ($87,930;$171,312) | | $3,133 ($-40;$12,041) |
| AE | $116,765 ($48,021;$168,659) | $129,748 ($62,053;$180,027) | | $-12,983 ($-25,629;$6,155) |
| **Radiation Benefit** | | | | |
| Life-years | 43.17 (26.11;57.59) | 39.43 (31.66;45.94) | | 3.74 (-5.58;12.42) |
| QALY | 12.08 (7.57;15.89) | 10.35 (8.31;12.03) | | 1.74 (-0.79;3.99) |
| Total Cost | $238,951 ($188,336;$264,971) | $247,834 ($184,194;$270,114) | | $-8,883 ($-21,454;$12,269) |
| PLGG | $122,213 ($88,119;$181,318) | $98,877 ($81,587;$128,356) | | $23,336 ($5,992;$54,653) |
| AE | $116,738 ($48,051;$168,649) | $148,957 ($85,648;$182,925) | | $-32,219 ($-70,892;$-972) |

**Supplementary Table 3:** Estimates of life-years after diagnosis, QALYs (Discounted) and Costs (Discounted), for fused patients, and fused patients who progressed.

**Supplementary Figures**

**Supplementary Figure 1:** Kaplan-Meier estimates of progression-free-survival and overall-survival for the SickKids institutional cohort.


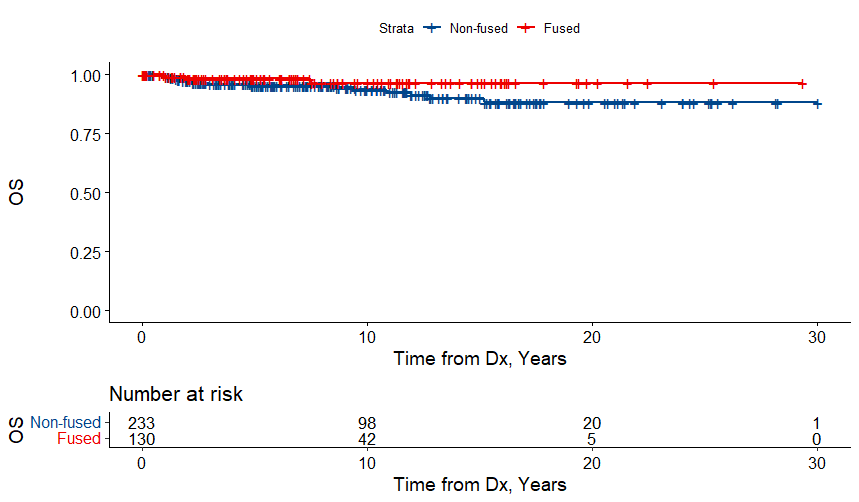


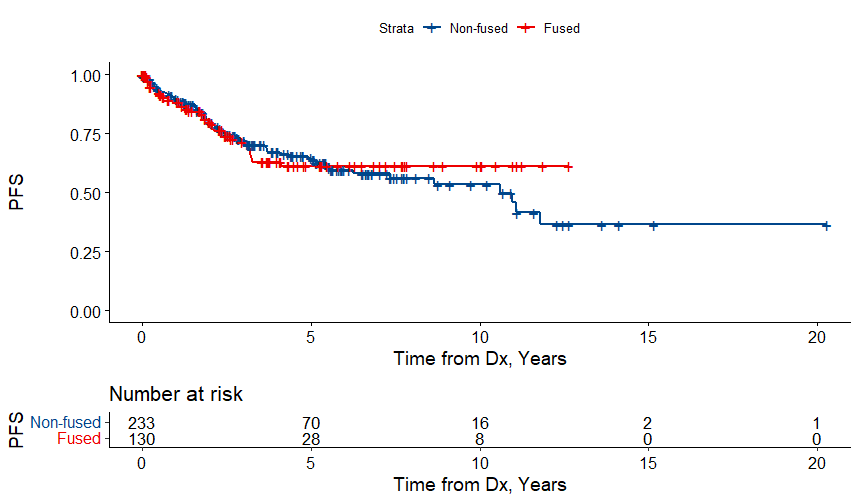


**Supplementary Figure 2:** Fitted transition probabilities for all model transitions.

**
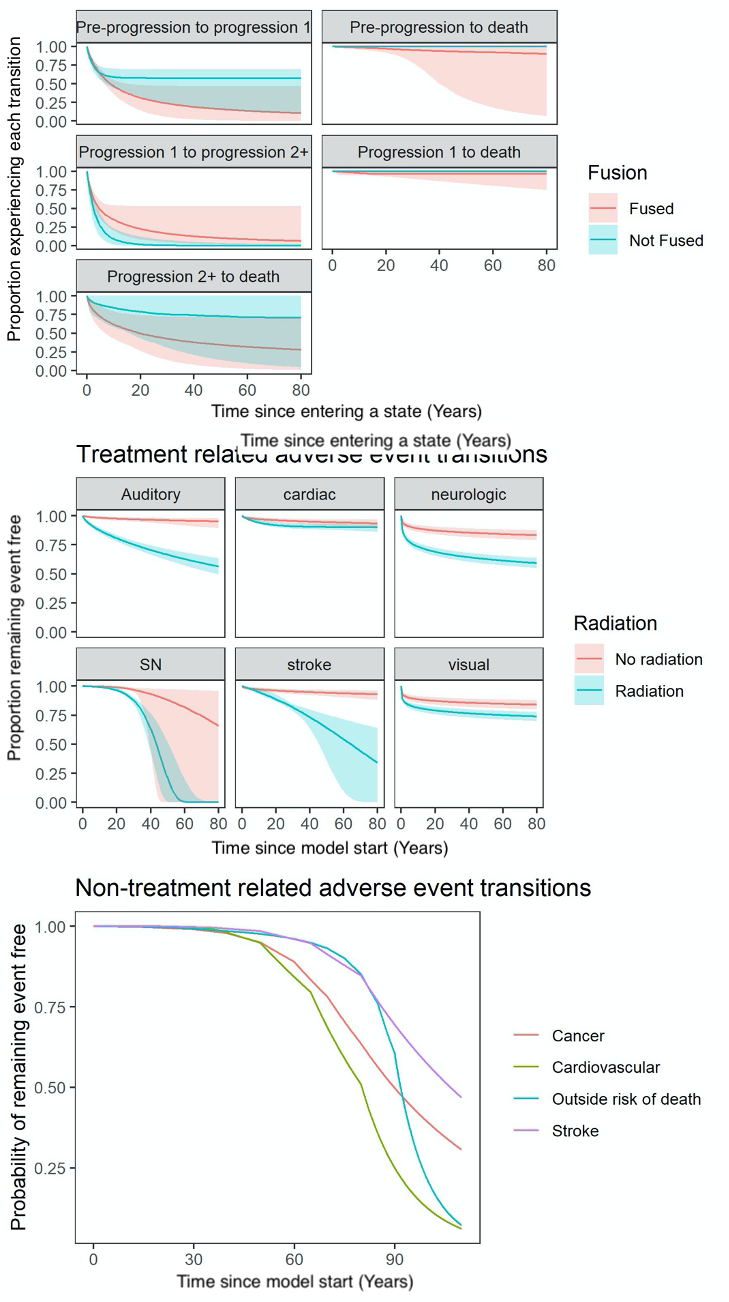
**

**Supplementary Figure 3:** Difference in cumulative incidence of all-cause mortality (intervention minus control) for all fused patients **(A & C)**, and for fused patients who progressed **(B & C)** under a no radiation benefit assumption (**A & B)** and a radiation benefit assumption (**C & D**).

**
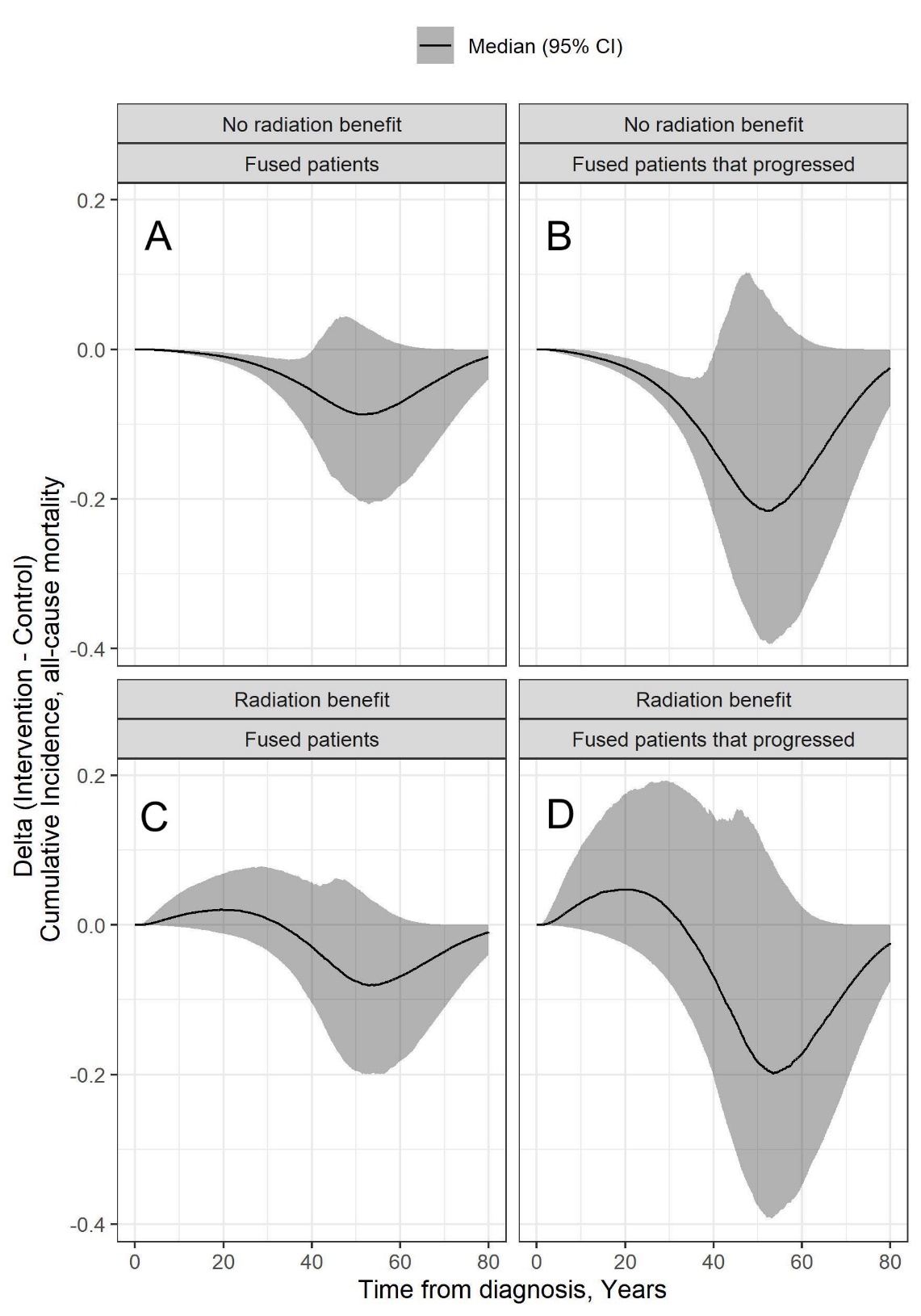
**

**Supplementary Figure 4:** Cumulative incidence of adverse events.

**
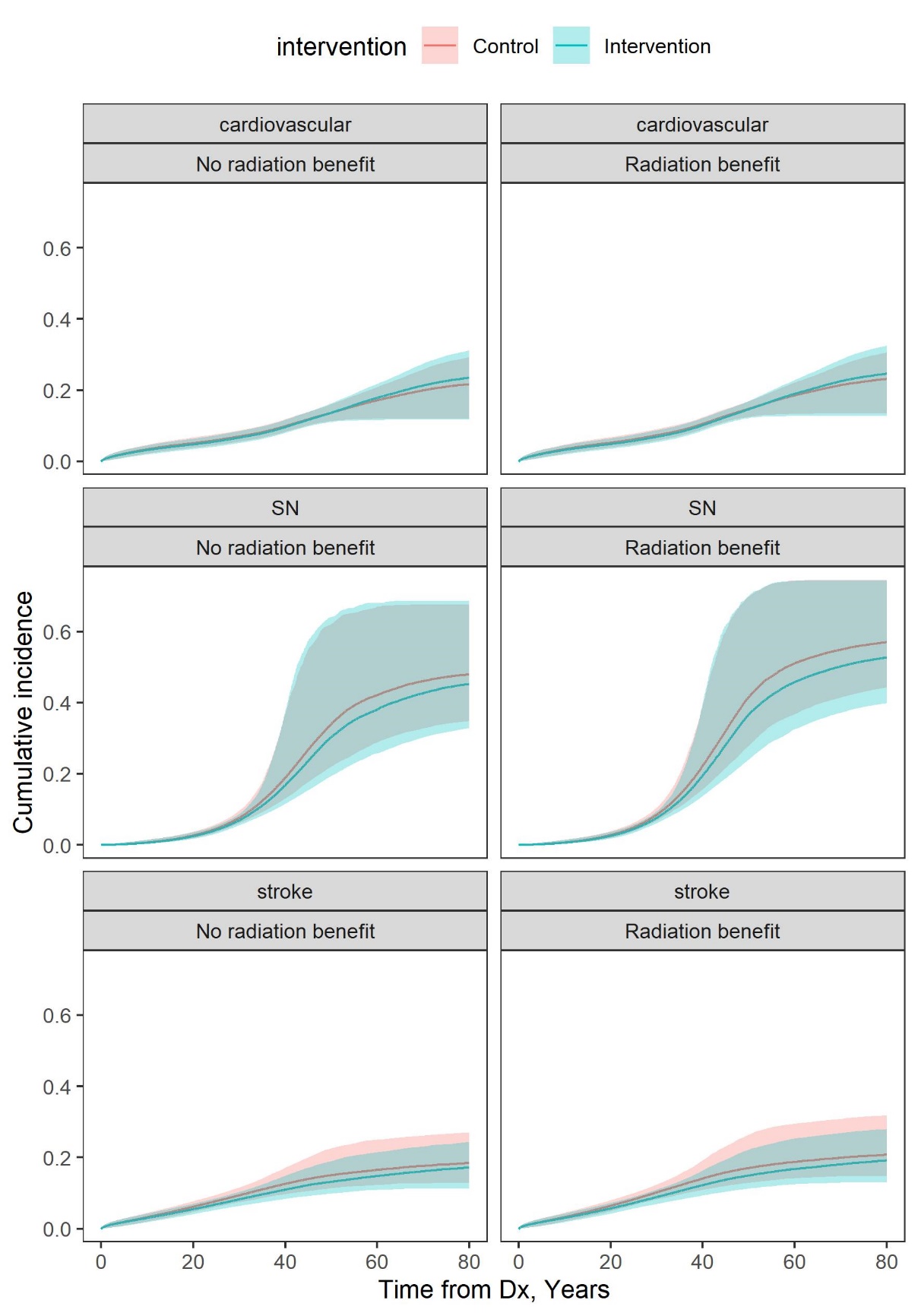
**

| **AE risk due to receiving radiation therapy** | **Costs** | | | **QALYS** | | |
| --- | --- | --- | --- | --- | --- | --- |
|  | **Intervention** | **Control** | **Delta** | **Intervention** | **Control** | **Delta** |
| **No radiation benefit** | | | | | | |
| No increased risk in AE | $220,201 | $220,067 | $134 | 12.62 | 12.62 | 0.00 |
| 10% of CCSS risk | $222,152 | $222,980 | -$828 | 12.29 | 12.17 | 0.12 |
| 20% of CCSS risk | $222,569 | $223,708 | -$1,139 | 12.10 | 11.91 | 0.19 |
| 30% of CCSS risk | $222,728 | $223,837 | -$1,109 | 11.96 | 11.72 | 0.25 |
| 40% of CCSS risk | $222,527 | $223,652 | -$1,126 | 11.84 | 11.55 | 0.29 |
| 50% of CCSS risk | $222,913 | $224,027 | -$1,114 | 11.74 | 11.42 | 0.33 |
| 60% of CCSS risk | $223,007 | $224,032 | -$1,025 | 11.65 | 11.29 | 0.36 |
| 70% of CCSS risk | $223,166 | $224,163 | -$997 | 11.58 | 11.18 | 0.39 |
| 80% of CCSS risk | $223,413 | $224,521 | -$1,107 | 11.50 | 11.09 | 0.42 |
| 90% of CCSS risk | $223,610 | $224,729 | -$1,119 | 11.44 | 11.00 | 0.44 |
| CCSS radiation risk | $223,811 | $225,041 | -$1,231 | 11.38 | 10.92 | 0.46 |
| **Radiation benefit** | | | | | | |
| No increased risk in AE | $222,099 | $221,783 | $316 | 13.64 | 13.83 | -0.18 |
| 10% of CCSS risk | $225,615 | $226,338 | -$723 | 13.16 | 13.19 | -0.04 |
| 20% of CCSS risk | $226,261 | $227,187 | -$927 | 12.87 | 12.82 | 0.05 |
| 30% of CCSS risk | $226,232 | $227,258 | -$1,026 | 12.67 | 12.56 | 0.11 |
| 40% of CCSS risk | $226,140 | $227,166 | -$1,026 | 12.50 | 12.34 | 0.16 |
| 50% of CCSS risk | $226,623 | $227,719 | -$1,095 | 12.36 | 12.15 | 0.20 |
| 60% of CCSS risk | $226,612 | $227,661 | -$1,049 | 12.23 | 11.99 | 0.24 |
| 70% of CCSS risk | $226,828 | $227,854 | -$1,026 | 12.12 | 11.85 | 0.27 |
| 80% of CCSS risk | $227,140 | $228,304 | -$1,164 | 12.03 | 11.73 | 0.30 |
| 90% of CCSS risk | $227,291 | $228,497 | -$1,205 | 11.94 | 11.61 | 0.33 |
| CCSS radiation AE risk | $227,309 | $228,538 | -$1,229 | 11.86 | 11.50 | 0.36 |

CCSS: Childhood Cancer Survivorship study. QALYs: Quality- Adjusted Life Years. AE: Adverse event risk.
All scenario analyses were done deterministically. No increased risk in AE assumes that individuals who receive radiation therapy have the same risk as those who received chemotherapy in the Effinger et al study. Delta = Intervention – Control.

**Supplementary Table 4:** Scenario analysis varying the risk of radiation related AE from no increased risk of AE due to radiation to the radiation risk sourced from the CCSS study.
